# A qualitative study on continuous deep sedation until death as an alternative to assisted suicide in Switzerland

**DOI:** 10.1186/s12904-021-00761-y

**Published:** 2021-05-14

**Authors:** Martyna Tomczyk, Nathalie Dieudonné-Rahm, Ralf J. Jox

**Affiliations:** 1 Institute of Humanities in Medicine, Lausanne University Hospital & University of Lausanne, Av. de Provence 82, CH-1007 Lausanne, Switzerland; 2 Palliative Care Unit, Geneva University Hospitals, Chemin de la Savonnière 11, 1245 Collonge Bellerive, Geneva, Switzerland; 3 Palliative & Supportive Care Service, Chair in Geriatric Palliative Care, Lausanne University Hospital & University of Lausanne, Av. Pierre-Decker 5, CH-1011 Lausanne, Switzerland

**Keywords:** Continuous deep sedation until death, Palliative sedation, Temporary sedation, Assisted suicide, Palliative care, Qualitative study, Physicians, French-speaking part of Switzerland

## Abstract

**Background:**

According to the European Association for Palliative Care, decisions regarding palliative sedation should not be made in response to requests for assisted dying, such as euthanasia or assisted suicide. However, several studies show that continuous deep sedation until death (CDSUD) – a particular form of sedation – has been considered as an alternative to these practices in some countries. In Switzerland, where assisted suicide is decriminalized and CDSUD is not legally regulated, no studies have comprehensively investigated their relation. Our study aimed to identify and describe the experience among palliative care physicians of CDSUD as a potential alternative to assisted suicide in the French-speaking part of Switzerland.

**Methods:**

We performed an exploratory multicentre qualitative study based on interviews with palliative care physicians in the French-speaking part of Switzerland and conducted linguistic and thematic analysis of all interview transcripts. The study is described in accordance with COREQ guidelines.

**Results:**

We included 10 interviews conducted in four palliative care units. Our linguistic analysis shows four *main* types of sedation, which we called ‘rapid CDSUD’, ‘gradual CDSUD’, ‘temporary sedation’ and ‘intermittent sedation’. CDSUD (rapid or gradual) was not considered an alternative to assisted suicide, even if a single situation has been reported. In contrast, ‘temporary’ or ‘intermittent sedation’, although not medically indicated, was sometimes introduced in response to a request for assisted suicide. This was the fact when there were barriers to an assisted suicide at home (e.g., when transfer home was impossible or the patient wished not to burden the family).

**Conclusion:**

These preliminary results can guide clinical, ethical, linguistic and legal reflection in this field and be used to explore this question more deeply at the national and international levels in a comparative, interdisciplinary and multiprofessional approach. They can also be useful to update Swiss clinical guidelines on palliative sedation in order to include specific frameworks on various sedation protocols and sedation as an alternative to assisted suicide. Potential negative impacts of considering palliative sedation as an alternative to assisted suicide should be nuanced by open and honest societal debate.

## Introduction

In palliative medicine, sedation is an important and necessary therapy for selected patients with otherwise refractory distress [1–3]. The European Association for Palliative Care (EAPC) defines this practice as “the monitored use of medications intended to induce a state of decreased or absent awareness (unconsciousness) in order to relieve the burden of otherwise intractable suffering” [1]. Sedation varies in duration and depth, it is qualified as brief, intermittent, or continuous and light, mild, or deep sedation [4]. The term ‘palliative sedation’ is general and encompasses all forms of sedation [1].

Continuous deep sedation until death (CDSUD) is a particular form of palliative sedation. Ethically and clinically, it should be considered as an exceptional therapy of last resort in very particular medical situations, when all other therapy (drug and non-drug treatments) has been ineffective, and only when the patient is in the very terminal stages of irreversible disease with an expected prognosis of hours or days [1, 3–5]. Concerning the depth of sedation, Morita et al. [6] propose two types of intervention protocol: 1) Gradual continuous deep sedation as a result of proportional sedation; and 2) Rapid continuous deep sedation to induce unconsciousness quickly, without proportionality. However, extensive literature clearly shows that it is not only the clinical practice of CDSUD, but also the terminology and definitions of this therapy that are heterogeneous and sometimes highly controversial. This problem has been frequently demonstrated and discussed in the international literature for several years, without consensus or guidelines being achieved [4, 5, 7–12]. In this paper, we use the framework of terms and conceptualizations proposed by the EAPC [1] and Morita et al. [6].

In palliative medicine, CDSUD is considered categorically distinct from assisted dying, such as euthanasia or assisted suicide. According to this understanding, decisions regarding sedation should not be made as a response to a request for assisted dying [1–5]. However, a qualitative study performed in the United States and the Netherlands indicates that justifications for this practice are not the same in different countries and may imply the wish to hasten death [13]. Empirical studies performed in the Netherlands and Belgium [14–18] clearly show that, in some cases, this type of sedation is considered as an alternative to euthanasia. For example, in the Netherlands, some physicians encourage patients to abandon their euthanasia wish and prefer rapid CDSUD which qualifies as ‘normal practice’ in the national guidelines [19] and involves less bureaucracy [20–22].

Issues associated with a possible relation between CDSUD and assisted suicide have scarcely been explored or discussed in the international literature. Reflections are essentially theoretical [23–28], without empirical exploration of the complex reality. Switzerland presents a useful opportunity to explore the above question in depth and from various perspectives.

In Switzerland, CDSUD is not regulated by law. In contrast, suicide assistance is decriminalized, without explicit legal regulation [29]. According to article 115 of the Swiss Penal Code, suicide assistance is a crime if and only if the motive is selfish. Article 115 does not require the involvement of a physician nor that the patient be in the terminal stages of illness [29]. This law is interpreted as legal permission for established organizations to assist in suicide. Contrary to the Netherlands, Belgium and Canada, but similar to those US states allowing suicide assistance [30], euthanasia is strictly prohibited in Switzerland [31]. Assisted suicide is socially accepted and represents 1–1.5% of all Swiss deaths annually, with a consistent increase over the last few years [32, 33]. At the epistemic and ethical level, it attracts particular attention [34].

The Swiss model of assisted suicide, called the ‘civic model’, is unique in the world [34–36]. It means that assisted suicide is largely seen as a civic rather than a medical act. It is provided by private right-to-die societies, *Exit Suisse Romande* in the French-speaking part of the country, for example. In this case, the physician’s role is limited to prescribing a lethal dose of the drug natriumpentobarbital and certifying the state of health and full decisional capacity of the patient concerned [37, 38]. Drug procurement and preparation are provided by volunteers from the right-to-die societies, who are often laypersons (but there are also some healthcare professionals who are active within these associations). If a physician is a volunteer of such an organization, he is present as a ‘private citizen’, not as a ‘physician’. Most commonly, patients self-administer the drug in their own home, but healthcare facilities also allow it [39]. Although assisted suicide is allowed in nursing homes and hospitals, the conditions for accessing it in the latter are rather restrictive.

A qualitative study performed by Gamondi et al. [40] shows that Swiss palliative care physicians have not received specific training in managing assisted suicide requests, although they regularly receive them. The study also shows that the majority of physicians explored the origins of the patient’s request and proposed alternatives. However, these alternatives are not clearly specified or discussed in the paper.

In June 2018, the Swiss Academy of Medical Sciences (SAMS) published new medico-ethical guidelines: “Attitude towards the end of life and death” [41]. This document lacks legal authority but offers safeguards for assisted suicide, as well as stricter limits than the law. It specifies that assisted suicide should remain the exception and invites healthcare professionals to propose alternatives to assisted suicide, such as stopping life-sustaining measures, ceasing eating and drinking, and administering a sedation. The document thus indicates ‘sedation’ as one alternative to assisted suicide, but it does not specify the form of sedation it refers to (e.g., mild sedation, temporary sedation, or CDSUD).

Thus, it is legitimate to wonder whether CDSUD is considered as an alternative to assisted suicide by health care professionals themselves. To the best of our knowledge, this topic has not been empirically explored in Switzerland so far.

The objective of this study was to identify and describe the experiences among palliative care physicians of CDSUD as a potential alternative to assisted suicide in the French-speaking part of Switzerland.

Contrary to Morita et al. [6], we did not specify the mode of administration of CDSUD due to the lack of international consensus on how to define and apply this type of sedation and because our goal was to explore in depth a reality that is, so far, unknown. This approach was decided at the time of designing this study.

## Methods

### Study design

We performed an exploratory multicentre qualitative study based on face-to-face interviews with physicians working (or who have worked) in palliative care units in the French-speaking part of Switzerland [42]. We conducted our study between February and November 2019 and describe it in the paper in accordance with the consolidated criteria for reporting qualitative research (COREQ) [43].

### Legal and ethical aspects

According to the Swiss Federal Act on Research involving Human Being, a review by an ethics committee was not required for our study because it was not of an interventional nature, did not involve the collection of personal health data and posed no risks to the participants. Participation was voluntary and the oral consent of each participant was systematically sought and obtained in all cases. Permission to audio-recording of the interviews was also obtained from each participant. Previously prepared information was sent by e-mail or delivered to all participants. The information mentioned that the name of the palliative care units may be indicated in publications resulting from this study but no material allowing identification of the participants. It was also clarified that the study was carried out as part of post-doctoral research by MT, who was, at the time, a post-doctoral researcher in medical ethics with nine years of experience in conducting qualitative research. Prior to this study, MT had no relationship (hierarchical, family, etc.) with the participants; in all cases, the research was the first contact. To ensure total confidentiality for the participants, only the main researcher (MT) had access to the recordings and integral transcripts; NDH and RJ had access to anonymised quotations selected by the main researcher.

### Inclusion process

The heads of palliative care units with the ‘palliative quality’ label awarded by the Swiss Association for Quality in Palliative Care in the French-speaking part of Switzerland were contacted by e-mail by MT. If the head of an unit agreed to participate in the study, he/she sent an e-mail to all physicians working in the palliative care unit or structure associated (e.g., mobile palliative care team) to inform them of the research. Next, physicians who volunteered to participate contacted the researcher and a date was arranged for a meeting. Progressive inclusion (inclusion until data saturation) was chosen. Data saturation was defined as the point at which no new themes emerged from the analysis of the material. The Comparative Method for Themes Saturation (CoMeTS) was used in order to achieve rigorous data saturation [44].

### Participant selection

Only three inclusion criteria were imposed: to be a physician who had worked or was working in a palliative care unit in the French-speaking part of Switzerland, who was available, and who gave consent to participate. No restrictions regarding gender, age, experience, diploma, current role and position in palliative care, or native language were set; our aim was to have a diverse population of physicians.

### Data collection

Data was collected by the main researcher between April and July 2019. Face-to-face interviews were conducted at a location chosen by the participants and this was always their workplace. The participant was alone with the researcher. Comprehensive information about the study was given at the beginning of each interview. The researcher explained that the study aimed to explore any experiences on a palliative care unit, not necessarily related to the current workplace. An interview guide based on a preliminary literature review and containing very general themes was used. The topics are listed in Table 1.

**Table 1:**
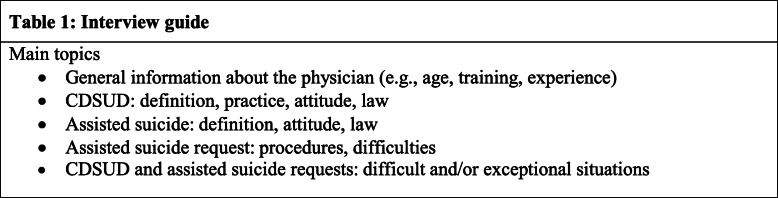
Interview guide

The themes were pilot tested with the first three participants, and as no substantive changes were made, they were included in the data set. Questions were also initiated by the interviewee’s answers. All interviews were conducted in French (the first language – native or learned – of all the participants and the researcher). Audio recording was used to collect the data and no notes were made, either during or after the interview. Interview recordings were manually transcribed, and the audio recording was deleted afterwards. The transcripts were returned to the participants for proofreading and validation. All participants validated their version. In order to ensure confidentiality to the participants, all identifying information was removed from these versions by the main researcher. The information removed was not essential and did not influence the analysis.

### Data analysis

We chose linguistic and thematic analysis with continuous theming (not using a framework) because of the lack of research on this topic and to enable us to explore our material in depth [45]. In other words, the majority of the themes were not identified in advance; they were inductively derived from the data. The data was analysed in three main steps: vertical analysis (interview by interview), transversal analysis (all interviews) and data triangulation. In the first step, a linguistic analysis of each interview transcript was performed by the main researcher and, sentence by sentence, semantic fields were gradually searched for and noted. Next, a theme was assigned to each semantic field. All themes were then grouped into central themes and sub-themes. Significant and lengthy quotations were extracted from the interview transcripts and associated with each central theme and sub-theme. In the second step, transversal analysis was conducted with the aim of identifying common key themes and sub-themes. Variations in theme by age, gender, experience, and qualifications were not examined. Finally, the results were discussed by all three researchers (MT, NDR and RJJ) in order to check the consistency of the resulting themes and to increase methodological reliability. Consensus was reached. All analysis was carried out on the original versions in French and only the results and quotations were translated into English by MT. In this article, only the main results and significant quotations are presented; no important aspects of the study and/or those directly related to the objective of the study have been omitted. Other aspects of the data will be presented in other papers.

**Fig. 1:**
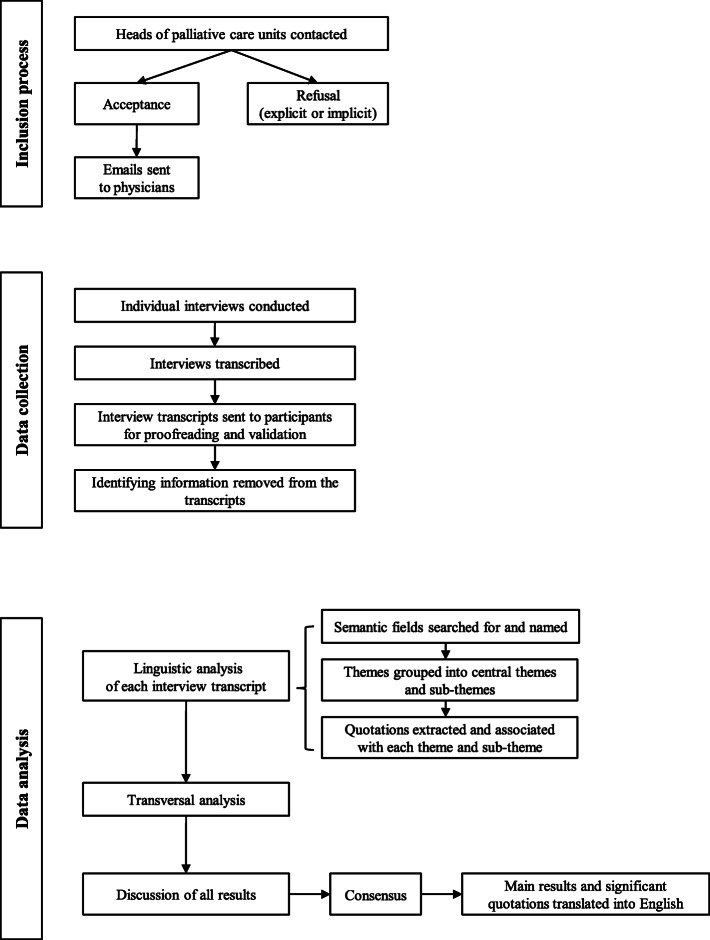
Methods

The quotations illustrate majority and minority experiences. In order to reinforce the anonymity of the participants, the number assigned to each interview during the analysis is not indicated here. Considering that the population is homogeneous as regards profession, and that correlations between age, experience, etc. were not researched, our approach has no impact on the interpretation of the results. The methods used in the study are summarized in Fig. 1.

## Results

### Sample size and characteristics

#### Palliative care units

**Fig. 2:**
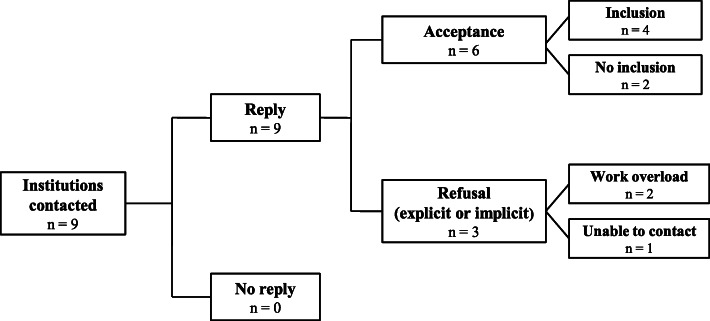
Sample size of palliative care institutions

In total, the researcher contacted nine palliative care units by e-mail, all of which replied. Six units agreed to participate in the study: four of them were included when data saturation was reached. The sample size is presented in Fig. 2.

#### Participants and interviews

**Fig. 3:**
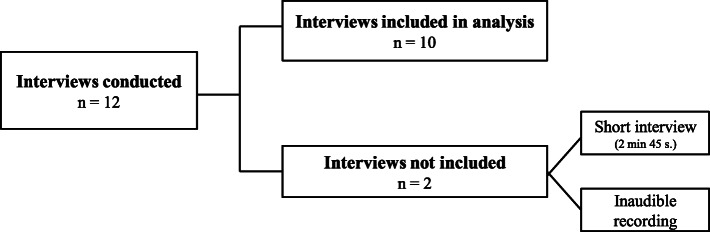
Sample size of interviews

The inclusion process was progressive. The sample size was determined by theoretical saturation. A total of 12 interviews were performed, 10 of which were transcribed and included in the analysis. The results presented below emerged from those 10 interviews. The interview sample is presented in Fig. 3.

The characteristics of the 10 participants whose interviews were included in the analysis are shown in Table 2.

**Table 2:**
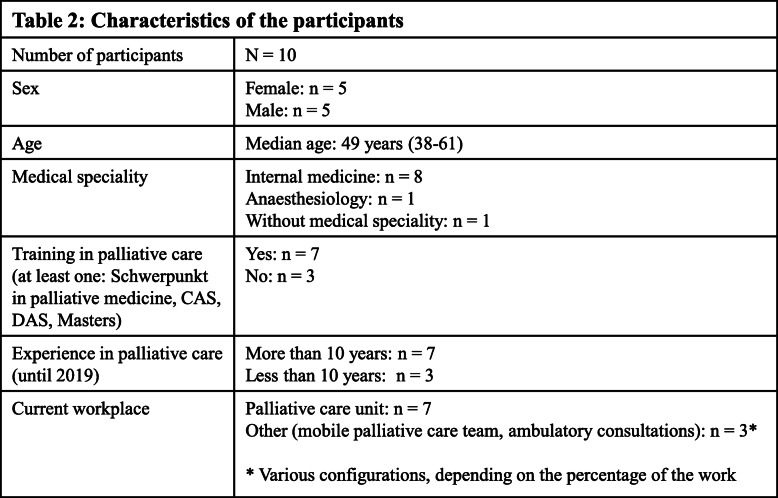
Characteristics of the paticipants

Interviews lasted between 13 and 46 min. The total duration of all interviews was 302 min (five hours), the median duration was 30 min.

### Findings

**Fig. 4:**
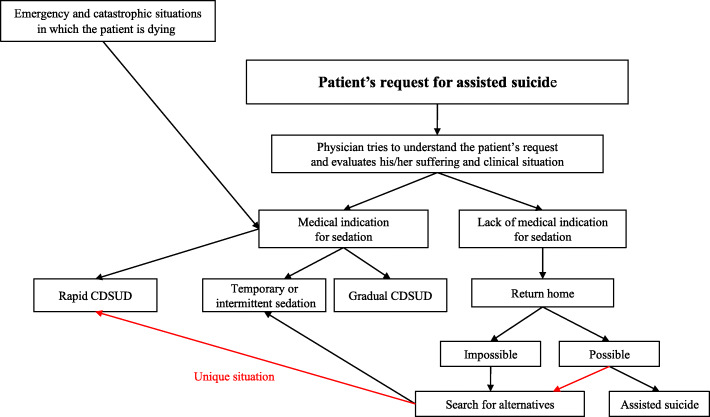
Summary of results

The results are shown schematically in Fig. 4 (see below).

#### Sedation in palliative medicine and assistance in suicide are different approaches

First, it should be clarified that all physicians (except one) clearly differentiated sedation in palliative medicine from assistance in suicide. They stated that contrary to assistance in suicide, sedation is a palliative treatment based on a medical indication.

One physician explained it as follows:

“[…] *they are two different things: in the first* [assisted suicide], *the person wants to end his life and in the other* [sedation] *the person is in a situation where nothing can be done for him, this is unbearable and we decide to induce a therapeutic treatment. So, we are squarely in another thing.”*

Another physician stated it in this way:

*“I think they are two completely different approaches: one is a life-sustaining therapeutic approach and the other is killing yourself. And these two different approaches must remain different*.*”*

In line with this, physicians were generally opposed to considering sedation as an alternative to assisted suicide. As one physician explained it:

*“We are not going to propose sedation in place of assisted suicide.* […] *I think that the proposal for sedation at the request of assisted suicide is a great drift.”*

Another physician illustrated it as follows:

*“I think that it is not an answer* [sedation in place of assisted suicide], *it is not A = B. In other words, it is not that the patient is talking about assisted suicide that we should think about sedation.* [...] *I don’t think we can say that as soon as the patient talks about assisted suicide, it should be assimilated to sedation because in this context sedation is a form of suicide or an alternative to suicide.”*

All participants reported that when a patient requests assistance in suicide, the first step they take is to engage in extended and repeated discussions with him or her, without judging the decision, in order to better understand the reasons for the request. Here is an illustration by one physician:

*“*[...] *it is necessary to welcome that* [this request], *to accompany, to understand what is hidden behind. Often it is desperation, it’s fear, a fear of suffering, a loss of meaning... this feeling of being a burden, this feeling of being useless... then this desire to disappear, this desire for it to be stopped, this need to be freed from a situation that is becoming unbearable.”*

In all cases, the participants try to improve the holistic care:

*“We try to take into account the depression, the pain, the financial and psycho-social problems, everything that concerns the whole family, the entourage. We try to do everything*.*”*

If, despite this, the patient’s wish for assisted suicide persists, most participants re-evaluate the clinical and personal situation. If the request for assisted suicide persists because of unbearable suffering, the physicians discuss the possibility of sedation. They clearly stated that sedation should only be introduced for medical reasons, not as an alternative to assisted suicide. However, they reported exceptional situations in which sedation, although *not* medically indicated, was introduced in response to an assisted suicide request. Our linguistic analysis showed four *main* types of sedation, which we called ‘rapid CDSUD’, ‘gradual CDSUD’, ‘temporary sedation’ and ‘intermittent sedation’. These types of sedation are presented in Table 3.

**Table 3:**
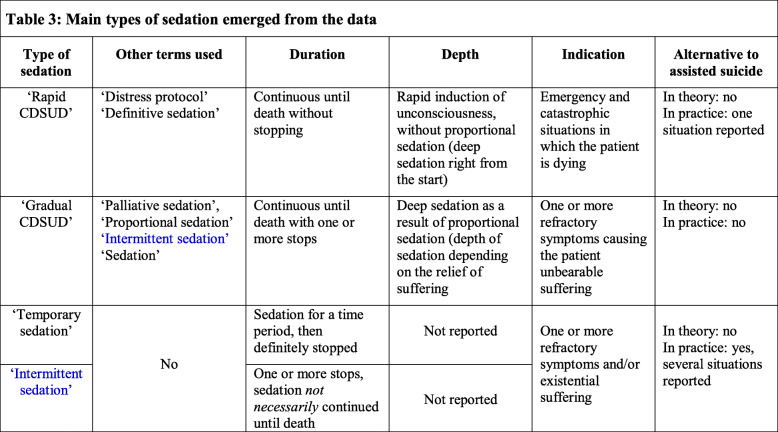
Main types of sedation emerged from the data

#### Rapid CDSUD is rarely considered as an alternative to assisted suicide

Most of the participants stated that sedation to induce unconsciousness rapidly, without proportional titration of drug dosage according to symptoms (in other words, deep sedation right from the start), and continuously until death without stopping – called ‘definitive sedation’ or ‘distress protocol’ – is only induced in emergency and catastrophic situations, such as a massive haemorrhage or asphyxia in dying patients. One physician stated:

*“They are emergency situations, truly irreversible situations for which we think we will have zero potential improvement to hope and in addition we must act quickly.* [...] *Yes, that is rather in emergency situations, when the patient is choking or bleeding. Similarly, if the patient is epileptic and resistant to all drugs, it doesn’t make much sense to wake him up and he re-convulses.”*

Another physician reported it as follows:

*“[...] someone is bleeding out. He is dead in 5 minutes. Continuous deep sedation until death is so required*[...].”

Participants pointed out that in all other medical situations, another type of sedation is chosen. As one physician explained:

*“In principle, when we are not in emergency, we induce sedation just to relieve, so the depth will depend on the relief. We always start with temporary sedation* [...]*.”*

Another participant added:

*“The fact of switching to a deep and permanent form of sedation also means straightaway imposing the definitive nature of the loss of contact. This seems difficult to manage in practice (managing with the team, the family, patient himself).”*

All participants explicitly stated that this type of sedation is never considered as an alternative to assisted suicide.

However, one participant reported a very exceptional situation: although rapid CDSUD (during the interview, the participant clearly stated that this was rapid CDSUD) was not medically indicated and going home would have been possible, it was initiated as a response to an assisted suicide request. This sedation lasted several days. The participant described the context:

*“It was four or five years ago. A patient who was 70 years old* [...] *was here* [in the palliative care unit] *for mental distress and for uncontrollable pain in the post-operative period. He wanted to call Exit, then he did the Exit procedures. At the same time, the pain was difficult to relieve, we had to quickly increase the doses. The next day, he was a little drowsy, he was less well and suddenly they (the team) stopped everything and then the pain came back much stronger. This patient was very demanding. Already before, he didn’t want to live* [...]*; for himself, it was not the quality of life as he imagined. So that was the reason he called Exit. And the pain just added what it took to really want to die. After stopping the analgesic drugs, suddenly, they tried to juggle the analgesia, but never succeeded, and they ended up sedating him. When I came back (I wasn’t there during that time; it was during the weekend), the patient was sedated.”*

The participant also clearly pointed out the type of sedation:

*“It was not intermittent sedation in order to see whether he could complete the Exit project... He was sedated during two or three weeks, which meant he was physically fine... So, there, I was wondering. For me, it was a kind of ... it was as the title of your study* [laughs].*”*

Apart from this one situation reported by a single participant, no similar situation was reported, despite explicit questioning during the interview.

#### Gradual CDSUD is not considered as an alternative to assisted suicide

Most of the participants stated that deep sedation as a result of proportional sedation (depth of sedation depending on the relief of suffering) up until death – called ‘palliative sedation’, ‘proportional sedation’, ‘intermittent sedation’ or ‘sedation’ – is administered to relieve one or more refractory symptom(s) causing the patient unbearable suffering (conceptualizations of ‘refractory symptoms’ and ‘suffering’ are not presented in this paper).^2^ The depth of this sedation always depends on the relief of suffering; it is not deep from the start. One physician explained it as follows:

“*There is not only one sedation. Sedation is really patient-focused. It is really done for every patient. There is no comparison.* [...] *There are sedations more or less deep. We have clinical tools (scales) to assess it, such as RASS scales. I never aim, from the start, to have RASS -3 or -4. My goal is to see if the person feels comfortable* *[...].”*

Another physician specified that

“*It is never immediately deep. In the end, the patient sleeps, it is true*.”

This sedation is intermittent and, in the last step, continued until death. The participant explained:

*“Until death... It is very common to say: ‘This person, we have already sedated him one night, two nights, three nights’, and then we see that when we wake him up, it is worse. Thus, we say ‘we will continue the sedation and it will be until death’.”*

Many participants insisted that a proposal of this type of sedation is always based on medical indication and not considered as an alternative to assisted suicide. Here are two examples of explanations:

*“No* [palliative sedation cannot be considered as an alternative to assisted suicide]. *If an intolerable symptom is the motive for an assisted suicide request,* [...] *we can offer sedation to relieve this symptom, but only for this objective.”*

*“If medical conditions are right, we can always propose palliative sedation, before going home and contacting Exit. In this case, sedation is not an alternative to assisted suicide, because it is induced for medical indications.”*

#### Temporary or intermittent sedation is sometimes considered as an alternative to assisted suicide

Most of the participants also talked about ‘temporary’ or ‘intermittent sedation’. Contrary to rapid or gradual CDSUD, these types of sedation are *not necessarily* continuous until death. They are also administered to relieve one or more refractory symptoms and/or existential suffering, and induced for a time period determined in advance, then definitely stopped (‘temporary sedation’) or continued, sometimes until death (‘intermittent sedation’). The physicians did not say anything about the depth of these forms of sedation. This aspect did not emerge from our data, so it is not known whether this sedation is proportional or deep from the start.

Participants pointed out that these types of sedation are often administered in response to appropriate medical indication. However, most participants also reported situations in which these forms of sedation, while *not* medically indicated, were introduced in response to an assisted suicide request. This was especially the case when there were barriers to assisted suicide at home (e.g., transfer home not being medically possible or the patient not wanting to traumatize the family). Three physicians explained:

*“*[...] *it happens. I’ve seen it. There are a lot of reasons why patients don’t carry out assisted suicide: ‘I don’t want to come back home, because my husband/wife and my children are there. And then, after that they will live in the house where I committed suicide’. It is very difficult. Other reasons: ‘My family doesn’t want me to do Exit because it’s against their values, because they don’t want it to traumatize them. So, I am looking for an alternative’. There are patients who are bedridden, they are not independent, they are hospitalized and then, they say: ‘I can’t go back home. Kill myself, yes, I want to do it. But I can’t go back home. Physically, it’s impossible for me’.* [...] *So, there are many reasons which make that (it comes from the patient himself or his family) that finally the option ‘assisted suicide’ is aborted*. [...] *As assisted suicide can’t be carried out, we naturally look for an alternative...”*

*“*[...] *on the one hand, yes, that’s a kind of alternative. I have already seen patients in the unit who would like assisted suicide and for various reasons it was not possible, and we say OK and finally we induced sedation. In this context, sedation is a kind of alternative to assisted suicide. These situations are not very common.”*

*“There are patients whose wish was clear. They were a member of Exit all their life, they are determined, they want to realize an assisted suicide but, unfortunately, the evolution of their disease has meant that they can’t go back home, they can’t swallow anymore, they are not conscious enough to swallow. Just because it’s too late doesn’t mean they don’t have the right to be relieved. So, there, in this context, sedation is* [...] *an alternative to assisted suicide* [...]*”*

Finally, one participant reported a specific and highly exceptional situation: intermittent sedation was administered as an alternative to assisted suicide because of the impossibility of the patient returning home; however, this sedation proved to be ineffective and the only possible solution was to perform assisted suicide in the palliative care unit as an exceptional case. He explained:

*“*[...] *the ultimate argument was: the impossibility of going home. She said: ‘we refuse to realize an assisted suicide in our house that we built, that we wanted, which was the object of our dreams... We refuse to leave that to our children as an inheritance’. She refused to do this at home and she said that for her it was ethically impossible. And it is true that we had no arguments to go against our own arguments.* [...] *This situation is very interesting because we proposed intermittent sedation to this patient. And then she said OK, why not.* [...] *But it went very badly. The dose was increased. Despite this, she woke up. The awakening was catastrophic. She felt very badly.* [...] *Assisted suicide was exceptionally realized here, in this palliative care unit.”*

## Discussion

Our exploratory study on the experience among palliative care physicians of CDSUD as an alternative to assisted suicide in the French-speaking part of Switzerland is the first of its kind and provides interesting and useful data for clinical research and practice, and for societal debate.

First, our linguistic analysis showed four *main* types of sedation. From the descriptions given by the participants, we called these forms of sedation ‘rapid CDSUD’, ‘gradual CDSUD’, ‘temporary sedation’ and ‘intermittent sedation’. However, the participants used various terms to describe their practices. Sometimes, the same term (‘intermittent sedation’) designated two different practices. This result is in accordance with the existing international literature, including guidelines in which consensus on how to name and define different types of sedation in palliative medicine is lacking, despite many efforts and proposals [4–12, 46, 47]. That is the source of much ambiguity and difficulty at the clinical and ethical levels and has made empirical studies and guidelines less easily comparable and interpretable.

Clinically, according to the EAPC guidelines [1], CDSUD can be indicated (without proportional drug dosing) in some situations, such as “intense suffering”, “suffering [that is] definitely refractory” or “catastrophic event”. However, participants in our study only reported a “catastrophic event” as an indication for this type of sedation.. For the other situations of “intense suffering” or suffering that was “definitely refractory”, participants preferred gradual CDSUD, with proportional drug dosing. This is in accordance with European Society for Medical Oncology (ESMO) [2] and Swiss guidelines [48]. However, it should be noted that in these three documents, many terms are used to refer to this type of sedation.

Switzerland has four official languages (German, French, Italian and Romansh), each with their own linguistic-geographical areas. National guidelines on palliative sedation issued in 2005 do not explicitly mention CDSUD [48]. More recent studies show that this type of sedation is practiced in each linguistic region [49–52]. In the French-speaking part of Switzerland, terminological and conceptual issues with regard to CDSUD from the palliative care physician’s perspective have not been precisely explored to date, although there has been some study on ‘palliative sedation’ related to this linguistic region of Switzerland [53–55]. In contrast, a study conducted in the German-speaking part of Switzerland suggests that physicians and nurses working in hospitals (in a palliative care unit, intensive care unit, general internal medicine, and oncology) used heterogeneous terminology to describe CDSUD. Most physicians and nurses referred to the term ‘palliative sedation’ or ‘terminal sedation’ but, outside specialized palliative care, several healthcare professionals used broader terminology, such as ‘symptom control’, ‘supportive care’, ‘comfort therapy’ or no specific term at all [52]. These interesting data cannot be compared with our data. According to linguistic theory, language is an important part of culture [56, 57] and some studies have revealed that in culturally polymorphic countries there are variations in medical end-of-life decisions and practices between linguistic regions [58, 59]. This has also been demonstrated by Swiss studies [51, 60]. Further research on the terms and definitions used by palliative care physicians in the French-speaking part of Switzerland is necessary in order to have a positive impact on research, discussion and the quality of care achieved with this practice.

Second, our study clearly showed that, except for the unique situation reported by one participant, CDSUD (rapid or gradual) is not considered as an alternative to assisted suicide in the French-speaking part of Switzerland. It also indicates that ‘temporary’ and ‘intermittent sedation’are sometimes introduced in response to requests for assisted suicide, without necessarily having a medical indication. However, the participants of our study did not specify the depth of sedation in these case. Ethically, although the proportionality of sedation is a highly complex notion, it is essential for rigorous discussion. Moreover, the participants did not precisely describe other aspects of these sedations (e.g., drugs used, modalities of administration and hydration). These elements would have made it possible to understand whether sedation was used to allow death to occur naturally or to hasten death. Further empirical research is needed to better understand this complex conceptual and clinical aspect.

It should be noted that ‘temporary’ or ‘intermittent sedation’ as an alternative to assisted suicide, if it is not deep, seems more ethically acceptable than CDSUD because, contrary to CDSUD, it does not generally end a person’s ‘biographical life’ (by ending the ability to interact with other people) and probably does not shorten her ‘biological life’ [4, 5, 61]. In other words, interactions with other people are still possible. Furthermore, if even ‘temporary’ or ‘intermittent sedation is deep, there is an element of reversibility: in theory, it is possible to wake the patient and evaluate his or her situation and wishes regarding sedation. However, sometimes, despite the desire and intention to wake the patient, the patient dies [6]. That is why ESMO recommends informing the patient about the possibility of death during this form of sedation [2]. This issue did not emerge clearly from our data. It would be interesting to know whether the physicians had experienced this situation and, if they had, how they managed it in the context of a prior request to assistance in suicide. Finally, it should be noted that ‘temporary’ or ‘intermittent sedation can be induced in patients with advanced incurable illness, but not necessary during the dying process. In contrast, CDSUD can be induced only when the patient is in the very terminal stages of irreversible disease with an expected prognosis of hours or days at most [1, 3–5]. According to Twycross [4, 5], if the patient is not imminently dying, CDSUD would be tantamount to ‘slow euthanasia’. It is possible that for this reason participants in our study have chosen ‘temporary’ or ‘intermittent’ sedation as an alternative to assisted suicide. This reveals that they seem to be rather reluctant to accept assisted suicide and try to offer ways to help the patient while circumventing what may be morally distressing to them.

The participants in our study reported that ‘temporary’ or ‘intermittent sedation’ can be considered in exceptional cases and applied as an alternative to assisted suicide *without* medical indication. According to the EAPC [1], the ESMO [2] and many national clinical practice guidelines [3, 19], including Swiss recommendations [48], sedation is only indicated for medical reasons when the professionals’ intention is to relieve patients’ refractory suffering as the option of last resort, not as a response to a request for assisted dying. However, in Switzerland, according to recent medico-ethical guidelines published by the SAMS, ‘sedation’ may be considered as one alternative to assisted suicide [41]. That has been confirmed (but not explored) by Gamondi et al. [40].

Hence, Swiss clinical guidelines on palliative sedation [48] may need to be updated in order to take up this discussion on sedation as a potential alternative to assisted suicide in accordance with the SAMS medico-ethical guidelines [41]. This would help palliative care physicians better manage these rare clinical situations, facilitate open team discussion, but also discussion with the patient and family, and offer the patient more personalized and appropriate care. Furthermore, this question should be discussed in the training for palliative medicine and care.

Third, our study also showed that the most frequently reported non-medical reason to practise ‘temporary’ or ‘intermittent sedation as an alternative to assisted suicide is the impossibility of carrying out assisted suicide at home (e.g., transfer home is impossible or because of the patient’s wish not to traumatize the family). According to the Swiss model of assisted suicide, this assistance is provided by private right-to-die societies at the patient’s home, but can also be realized in hospital. However, as the conditions for accessing assisted suicide in hospitals are often restrictive and the process is cumbersome, the effect produced on teams could be to facilitate, more or less consciously, access to sedation despite the absence of any non-debatable indication [62, 63].

Belgian and Dutch physicians have reported using CDSUD for patients who had requested euthanasia when the requirements for euthanasia could not be met (i.e., a lack of capacity or too little time to consult a second physician) or because of moral reasons on the part of the physicians and the patient’s preferences [14]. This result cannot be compared with our findings because of the different systems of assisted dying in Benelux countries and Switzerland. However, lack of capacity is an interesting element to discuss in the context of Switzerland because a patient who requests assistance with suicide must have full decisional capacity and the physician’s role is to certify this [34–38]. It would be interesting to know whether Swiss physicians propose ‘temporary’ or ‘intermittent sedation when patient do not have full decisional capacity and thus cannot access assisted suicide. This element did not clearly emerge from our study and it would be desirable to conduct further study to explore this issue. Furthermore, international and comparative research on how physicians from foreign countries with diverse jurisdictions on end-of-life practices respond to requests for suicide assistance is necessary. It is particularly interesting in the context of France – a country that is unique in the world for CDSUD being explicitly and precisely regulated at the legal [64, 65] and clinical [66] levels, and assistance in suicide is strictly prohibited. It could help identify and better understand the cultural specificities of this question.

### Methodological limitations of the study

Several methodological limitations should necessarily be taken into account when interpreting this study and planning future research.

First, this was a qualitative study, therefore it cannot be used to verify hypotheses [67]. Our goal was to explore the field in order to reveal different aspects of a very complex and largely ignored reality. The qualitative design permitted an in-depth exploration of this reality from the physician’s perspective and comprehension of the relation between CDSUD and assisted suicide. Our results may be useful to develop a quantitative study at the regional and/or national level, and even from an international perspective.

Second, our sample size may appear relatively small: four palliative care units were included, 12 interviews conducted and 10 analysed. However, in the literature, consensus regarding sample size is lacking and considerations are highly heterogeneous in this area [68]. Our sample size was sufficient to reach theoretical saturation. The reality was explored in depth via the individuality of each participant. This approach corresponds to the principle of exploratory qualitative study; the objective is not to have a representative sample, but one that is socially significant [69].

Third, all the interviews were conducted in French and all analyses were carried out on the original versions of the transcripts. That might have reduced the risk of interpretive bias. The results and quotations were translated into English. Despite training in translation and sound knowledge of the two languages, the risk of semantic bias between French and English remains; translation is always approximate, whatever the strategy applied [70].

Finally, it should be noted that there was a gap between the interviews performed and the interviews included in the analysis. Indeed,the transcripts were returned to the participants for proofreading and validation and only validated interviews were included in the analysis. Therefore, passages concerning delicate issues (e.g., psychological suffering) had often been deleted from the validated version or had been modified. This had an impact on the results obtained and presented in this paper.

## Conclusion

CDSUD as an alternative to assisted suicide has not been empirically explored to date. Our original qualitative study, based on interviews with physicians in the French-speaking part of Switzerland, is the first of its kind on this topic. It shows that CDSUD is generally not considered as an alternative to assisted suicide; however, ‘temporary’ or ‘intermittent’ sedation is sometimes considered as such. These preliminary results can guide clinical, ethical, linguistic and legal reflection in this field and be used to explore this question more deeply at the national and international levels in a comparative, interdisciplinary and multiprofessional approach. In addition, the results could be useful to update Swiss clinical guidelines on palliative sedation issued in 2005, in order to include specific frameworks on this practice as an alternative to assisted suicide in accordance with the SAMS medico-ethical guidelines. Potential negative impacts of considering sedation as an alternative to assisted suicide should be nuanced by open and honest societal debate.

## Data Availability

Integral data are not available because they contain information that potentially permits the participants to be identified, particularly as the names of the institutions that participated in this study and the characteristics of the participants are explicitly indicated in this paper. Global data and quotes in French are available on request from the first author [MT], subject to institutional and participant consent.

## Abbreviations

CDSUD: Continuous deep sedation until death
EAPC: European Association for Palliative Care
ESMO: European Society for Medical Oncology
SAMS: Swiss Academy of Medical Sciences
